# Chemical Composition and *In Vitro* Bioaccessibility of Antioxidant Phytochemicals from Selected Edible Nuts

**DOI:** 10.3390/nu11102303

**Published:** 2019-09-27

**Authors:** Jazmín C. Stevens-Barrón, Laura A. de la Rosa, Abraham Wall-Medrano, Emilio Álvarez-Parrilla, Roberto Rodríguez-Ramirez, Ramón E. Robles-Zepeda, Humberto Astiazaran-García

**Affiliations:** 1 Instituto de Ciencias Biomédicas, Universidad Autónoma de Ciudad Juárez, 32310 Ciudad Juárez, Mexico; jazmin_stev@hotmail.com (J.C.S.-B.); ealvarez@uacj.mx (E.Á.-P.); 2 Departamento de Biotecnología y Ciencias Alimentarias, Instituto Tecnológico de Sonora, 85000 Ciudad Obregón, Mexico; roberto.rodriguez@itson.edu.mx; 3 Departamento de Ciencias Químico-Biológicas, Universidad de Sonora, 83000 Hermosillo, Mexico; robles.zepeda@unison.mx; 4 Coordinación de Nutrición, Centro de Investigación en Alimentación y Desarrollo, 83304 A.C. Hermosillo, Mexico; hastiazaran@ciad.mx

**Keywords:** tree nuts, peanuts, tocopherols, tocotrienols, phenolic compounds, flavonoids, tannins, bioaccessibility

## Abstract

The ultimate health benefits of peanuts and tree nuts partially depend on the effective gastrointestinal delivery of their phytochemicals. The chemical composition and in vitro bioaccessibility of tocopherols, tocotrienols and phenolic compounds from peanuts and seven tree nuts were evaluated by analytical and chemometric methods. Total fat and dietary fiber (g 100 g^−1^) ranged from 34.2 (Emory oak acorn) to 72.5 (pink pine nut; PPN) and from 1.2 (PPN) to 22.5 (pistachio). Samples were rich in oleic and linoleic acids (56–87 g 100 g^−1^ oil). Tocopherols and tocotrienols (mg·kg^−1^) ranged from 48.1 (peanut) to 156.3 (almond) and 0 (almond, pecan) to 22.1 (PPN) and hydrophilic phenolics from 533 (PPN) to 12,896 (Emory oak acorn); flavonoids and condensed tannins (mg CE.100 g^−1^) ranged from 142 (white pine nut) to 1833 (Emory oak acorn) and 14 (PPN) to 460 (Emory oak acorn). Three principal components explained 90% of the variance associated with the diversity of antioxidant phytochemicals in samples. In vitro bioaccessibility of tocopherols, tocotrienols, hydrophilic phenolics, flavonoids, and condensed tannins ranged from 11–51%, 16–79%, 25–55%, 0–100%, and 0–94%, respectively. Multiple regression analyses revealed a potential influence of dietary fiber, fats and/or unsaturated fatty acids on phytochemical bioaccessibility, in a structure-specific manner.

## 1. Introduction

Peanuts and tree nuts are considered natural functional foods. Besides being moderate-to-good sources of dietary fiber, macro (e.g., fats, protein) and micronutrients (e.g., magnesium, selenium and vitamins B), they contain a wide range of hydrophilic and lipophilic phytochemicals with health-promoting properties including sterols, tocols [tocopherols (T; α, β, γ, δ) + tocotrienols (T3; α, β, γ, δ)], unsaturated fatty acids, and phenolic compounds. The antioxidant and epigenetic effects of tocols regulate key events in lipid metabolism, inflammation, immunity, angiogenesis, cancer, and tumor metastasis [1,2], while monomeric (e.g., flavonoids) and polymeric (e.g., condensed tannins) phenolic compounds exert anti-inflammatory, anti-adiposity, and anti-cancer actions [3,4]; however, their individual and complementary bioactivity partially depends on their pharmacodynamics (systemic bioavailability), gastrointestinal (GI) bioaccessibility (amount released from food matrices), and first-pass bioavailability (enteral absorption and biotransformation) [5,6]. 

The amount of tocols released from food matrices in the GI tract depends on several physical and chemical factors—their interaction with macromolecules (e.g., pancreatic enzymes, fatty acids, and mucin) and their own physicochemical properties (e.g., hydrophobicity and polar surface area) are just two factors [5]. Reboul et al. [7] reported that the bioaccessibility of αT ranges from 0.5% (from apples) to 100% (from lettuce) passing by 11–14% from almonds and hazelnuts, while O’Callaghan and O’Brien [8] also reported a bioaccessibility of 11% for αT from apple sauce. The chemical interaction of tocols with fatty acids [saturated (SF), mono (MUFAs) and polyunsaturated (PUFAs)], the food matrix’s particle size and macronutrient composition and their interaction with GI enzymes affects tocols’ bioaccessibility [9,10]. 

Hydrophobic forces and molecular hindering mechanisms play major roles on the in vitro bioaccessibility of lipophilic phenolic compounds (e.g., phenetyl phenolic acid esters and phenolic acid lactones), while hydrogen bonding and ionic forces are involved in the bioaccessibility of hydrophilic ones [6]. Ortega et al. [11] reported that high-fat food matrices favor the bioaccessibility of phenolic compounds regardless of their degree of hydrophobicity, although this fact has been scarcely studied in peanuts and tree nuts. Mandalari et al. [12] evaluated the bioaccessibility of phenolic compounds from pistachios (raw, roasted, and salted) under simulated GI conditions, reporting their highest bioaccessibility at low pH (gastric phase). It is noteworthy that besides fat (oil), certain tree nuts are particularly rich in total dietary fiber which prevents the bioaccessibility of phenolic compounds in the upper GI tract but not under colonic conditions where they are fermented by the resident microflora [6,13]. 

The above-mentioned evidence supports the idea that the chemical nature and molecular organization of nut matrices define the bioaccessibility and luminal-to-systemic bioavailability of their phytochemicals. However, the extent to which a particular matrix component (e.g., total dietary fiber or fatty acids) positively or negatively influences the bioaccessibility of specific phenolic compounds (lipophilic and hydrophilic phenolic compounds, flavonoids and condensed tannins) and tocols (T + T3) has been scarcely studied. To our knowledge, the simultaneous evaluation of the in vitro bioaccessibility of all these antioxidant phytochemicals from peanuts and tree nuts is reported for the first time.

## 2. Materials and Methods 

### 2.1. Samples and Chemicals

Peanuts and seven tree nuts (raw) were used in this study. Almonds (ALM; *Prunus dulcis*) and walnuts (WNT; *Juglans regia*) were from Blue Diamond Growers (Sacramento, CA), white (WPN) and pink (PPN) pine nuts (*Pinnus cembroides*) and peanuts (PNT; *Arachys hypogaea*) were harvested in central Mexico (Hidalgo), pistachios (PIS; *Pistacia vera*) and pecans (PEC; *Carya illinoinensis*) were obtained locally (Valley of Juárez, Chihuahua México), and Emory oak acorns (EOA; *Quercus emory*) were collected from wild trees located in Cuitaca, Sonora Mexico (31°00′17″ N, 110°29′33″ O) during fruiting season (Jun-Jul). All samples were shelled, dehulled (inner skin), grounded, vacuum sealed and stored at −20 °C until use. 

Pure (≥93%) standards (β-carotene, (+)-catechin, fatty acid methyl esters (Supelco^®^ 37 component FAME mix), gallic acid, tocols (T, T3; α, β, γ, δ)], Folin–Ciocalteau phenol reagent, 4-(dimethylamino) cinnamaldehyde (DMAC) reagent, enzymes (e.g., pepsin, pancreatin), bovine bile, Analytical-grade salts (e.g., KOH, NaOH) and acids (e.g., H_2_SO_4_) were purchased from Sigma-Aldrich-Fluka (St. Louis, MO, USA). High-performance liquid chromatography (HPLC)- and analytical-grade solvents (hexane, methanol, acetone, and isopropyl and isobutyl alcohol) were obtained from JT-Baker (Avantar performance materials S.A. de C.V., Mexico). Tocomin^®^ SupraBio was obtained from Healthy Origins^®^ (Pittsburgh, PA, USA). 

### 2.2. Phytochemical Composition

#### 2.2.1. Chemical Composition 

Physicochemical analyses of all raw samples were carried out in accordance with the Association of Official Agricultural Chemists (AOAC) international official methods [14]: moisture (925.40), protein (950.48), fat (948.22), ash (923.03), total carbohydrate (by difference) and total dietary fiber (991.43 and AACC 32-07.01).

#### 2.2.2. Sample Fractionation 

**Figure 1:**
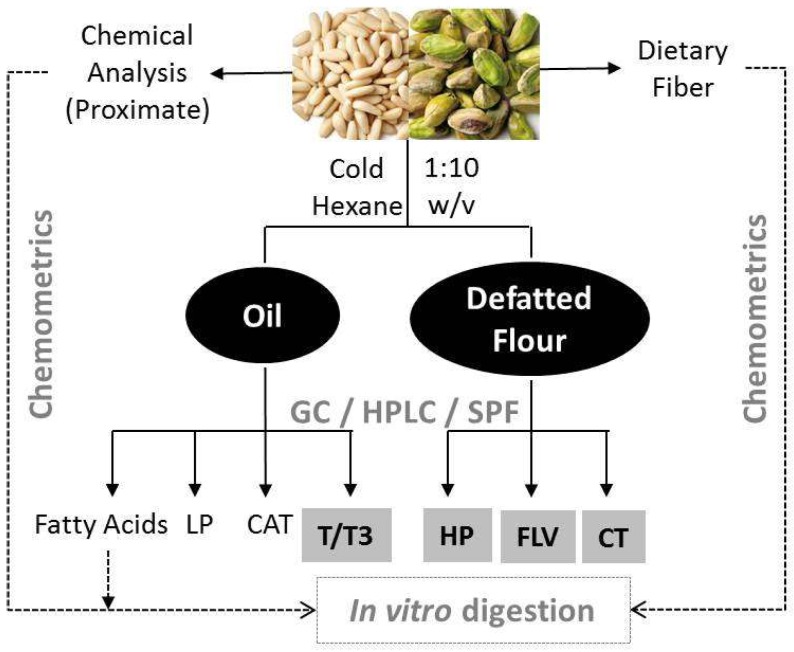
Experimental design. See text for experiment description. Methods: Gas chromatography (GC), high-performance liquid chromatography (HPLC) or spectrophotometry (SPF); Analytical assays: Carotenoids (CAT), lipophilic (LP) and hydrophilic (HP) phenolic compounds, tocols (T/T3), flavonoids (FLV), and condensed tannins (CT).

Oil extraction from samples was carried out according to Miraliakbari and Shahidi [15]. Briefly, each sample was homogenized in hexane (1:10 w/v), stirred for 3 min, and filtered (Whatman # 4 filter paper) under vacuum (Figure 1). 

The residue was re-extracted and both supernatants were mixed and dehydrated with anhydrous sodium sulfate (1 g). Sodium sulfate and hexane were removed by filtration and rotary evaporation at 40 °C, respectively. The oil was weighed, transferred to an amber bottle and sealed under nitrogen, while defatted flours (Figure 1) were left to dry overnight at room temperature in the dark to eliminate residual hexane. Both samples were kept at −80 °C until analysis.

#### 2.2.3. Fatty Acids

Fatty acids in edible oils were analyzed by gas chromatography (GC) as FAME, according to Isbell et al. [16]. Briefly, 0.25 mL of KOH (0.5 M) in methanol was added to 1 g of oil and incubated at 60 °C for 1 h. Then, 0.25 mL of H_2_SO_4_ (1 M in methanol) was added to this and incubated for another 15 min at 60 °C; finally, 0.25 mL of a saturated saline solution (NaCl) + 1 mL of hexane was added, before allowing the mixture to stand for two-phase separation (FAMEs were recovered in the upper layer). Quantification was performed as described by Núñez-Gastélum et al. [17] with certain modifications. The equipment consisted of a gas chromatograph 3800 (Varian Inc., Palo Alto, CA), with a flame ionization detector and a capillary column CP7485 88 (25 mm, 0.32 mm i.d. and thickness). Running conditions were: injection volume (1 µL), gas carrier (helium, 0. 6 mL·min^−1^), constant detector temperature (250 °C), and column temperature (50 °C for 1 min, 220 °C at a rate of 4 °C min^−1^ for 1 min, and 240 °C, maintained for 5 min). Quantification of FAME was achieved by comparing the area under the curve of each peak with those of pure standards and expressed as grams per one hundred grams of oil (g 100 g^−1^ oil). 

#### 2.2.4. Tocols (T + T3) 

A total of 60 mg of edible oil was mixed with 1 mL of hexane (HPLC grade), filtered with syringe membrane filters (Nylon, 0.45 µM) and placed in HPLC vials wrapped in aluminum foil. The equipment for tocol identification and quantification consisted of HPLC equipment (Perkin Elmer model 200 series), a LiChrosorb Si 60 (5 μM, 25 × 0.4 cm) normal-phase column and a fluorescence detector at 285 nm excitation and 325 nm emission wavelengths and the experimental conditions were as follows: isocratic, the glow rate was 1.0 mL·min^−1^, isopropanol:hexane (0.9:99.1 *v*/*v*) as mobile phase. T and T3 peaks were identified by comparing their retention time to that of pure standards (αT, αT3, γT, γT3, δT) and Tocomin^®^ (SupraBio, Healthy Origins^®^, Pittsburgh, PA, USA), a commercially available red palm oil extract rich in T3 (40%), αT (11%) and olein, which contains large amounts of αT3, βT3, γT3 and δT3 [18]. Results were expressed in mg of tocols.kg^−1^ of raw sample. The contents of βT3 and δT3 were quantified using the standard curves of γT3 and δT, respectively.

#### 2.2.5. Carotenoids

Sample processing was performed in the dark to avoid carotenoid degradation. Carotenoid (CAT) content was quantified with a UV-Vis spectrophotometer (BioRad Benchmark Plus, Bio-Rad Laboratories, Inc., Hercules, CA, USA) according to the method reported by Franke et al. [19], with some modifications. Oil samples (500 mg) were added to 2 mL acetone and the absorbance was read at 445 nm. Total carotenoids were determined using the following equation, and expressed as beta-carotene equivalents (*β*CE):

*β*CE (mg 100 g^−1^) = (*A* * mL * 10^6^)/(1000 * g * 2500) (1)

where: *A* = absorbance at 445 nm, mL = volume of the extracting solution (2 mL), g = sample’s weight (0.5 g), and 2500 = average absorption coefficient of a carotenoid molecule.

#### 2.2.6. Total Lipophilic and Hydrophilic Phenolic Compounds 

Lipophilic phenolic compounds (LP) were quantified in edible oils according to Berker et al. [20]. Briefly, Folin–Ciocalteu phenol reagent (1:10 *v*/*v*) and oil samples (30 mg·mL^−1^) were diluted in isobutyl alcohol. A volume of 25 mL of each solution was mixed in 100 mL of aqueous sodium hydroxide (0.1 M) and incubated at room temperature for 3 min. A volume of 125 mL of Folin–Ciocalteu reagent was then added, and the mixture was incubated for 15 min at 50 °C in the dark. The reaction was read at 665 nm in a UV-Vis microplate reader (BioRad Benchmark Plus). Gallic acid in isobutyl alcohol was used to build a standard calibration curve (0.006 to 0.2 mg·mL^−1^ in methanol; R^2^ > 0.95), and results were expressed in mg of gallic acid equivalents per 100 g (GAE.100 g^−1^) of raw sample.

To quantify hydrophilic phenolic compounds (HPs), one g of each defatted flour was mixed with 10 mL of aqueous acetone (80%, *v*/*v*) at room temperature and shacked for 10 min in an ultrasonic bath (Fisher Scientific FS220H, Thermo Fisher Scientific, Waltham, MA, USA). Afterward, the mixture was centrifuged at 3000 rpm for 10 min at 4 °C (Eppendorf 5810R, Eppendorf Hamburg, Germany). The supernatant was recovered, and the sample was subjected to one more extraction process, following the same conditions. The supernatants were combined, and the solvent was removed by rotary evaporation (40 °C). The extract was then frozen, freeze-dried (LABCONCO Freezone 6, Labconco, Kansas City, MO, USA) and further homogenized in methanol (5 mg·mL^−1^). The content of hydrophilic phenolic compounds was quantified using Folin–Ciocalteu reagent diluted in distilled water (ratio of 1:10 *v*/*v*). Absorbance (at 760 nm) was read in a UV-Vis microplate reader. Results were expressed as indicated for lipophilic phenolic compounds.

#### 2.2.7. Flavonoids and Condensed Tannins 

Total flavonoids were quantified in the same alcoholic extract used for the quantification of hydrophilic phenolic compounds. Briefly, 31 µL of the sample was mixed with 125 µL of distilled water and 9.3 µL of 5% sodium nitrite (*w*/*v*) and incubated for 5 min at room temperature; 9.3 µL of 10% aluminum chloride (*w*/*v*) was then added and incubated at room temperature for 3 min; lastly, 125 μL of 0.5 M sodium hydroxide was added to the mixture, and the mixturewas read at 510 nm in a microplate reader, using catechin as a standard (0.06 to 2 mg·mL^−1^; R^2^ > 0.95). Results were expressed as milligrams of catechin equivalents per 100 g (mg CE.100 g^−1^) of dry sample. Condensed tannins were quantified in the same extract according to Seeram et al. [21] in the same MeOH extract used for hydrophilic phenolic compounds and total flavonoids: 50 μL of the extract was mixed with 250 μL of DMAC reagent (0.1% *v*/*v*) in acidified methanol (10% HCl, *v*/*v*). The reaction was incubated for 5 min at room temperature, and the absorbance was read at 640 nm in a UV-Vis microplate reader. Results were expressed as indicated for flavonoids.

### 2.3. In Vitro Bioaccessibility 

#### 2.3.1. Static In Vitro Digestion Method 

The in vitro bioaccessibility of T, T3, flavonoids and condensed tannins was evaluated using the static in vitro digestion method reported by Olivas-Aguirre et al. [22] with minor modifications. *Oral phase.* Three healthy volunteers, who had their last meal at least 90 min prior to the test, were invited to the study and they provided written consent before participation (Project ID code (FO-CIP-01/254063), approval date (July 30th, 2016), Institutional review board (Biomedical Sciences Institute, Autonomous University of Ciudad Juarez)). After brushing their teeth without toothpaste, participants chewed 6 g of each crushed sample, 15 times for 60 s. Chewed samples were collected in a beaker, and subjects rinsed their mouths two times with 15 mL of distilled water for 30 s. Individual samples were adjusted to a final volume of 40 mL, transferred to a falcon tube and centrifuged at 3200 rpm at 37 °C for 15 min and the aqueous phase was separated. All individual samples (n = 3) were pooled, and the content of each phytochemical released at this phase was quantified. *Gastric phase.* The solid residue of the oral phase (bolus) was mixed with 40 mL of distilled water and the pH adjusted to 2 to be mixed with 55 mg of a solution pepsin from porcine gastric mucosa (≥ 250 units per mg protein) in 0.94 mL of 20 mM HCl, and incubated for 2 h at 37 °C. Subsequently, the sample was centrifuged at 3200 rpm at 37 °C for 15 min. *Intestinal phase.* A pancreatic enzyme solution was prepared 30 min before use, dissolving 3 mg of bovine bile (Sigma-Aldrich CAS: 8008-63-7) and 2.6 mg of pancreatin from porcine pancreas (Sigma-Aldrich 89 USP) in 5 mL of Krebs–Ringer buffer (118 mM NaCl, 25 mM NaHCO_3_, 11 mM glucose, 4.7 mM KCl, 2.5 mM CaCl_2_, 1.2 mM MgSO_4_, and 1.2 mM KH_2_PO_4_, pH 6.8). A volume of 5 mL of this solution was added to each sample coming from the gastric phase, pH was adjusted to ~7.3 and mixtures were incubated for 2 h at 37 °C. Lastly, the mixture was centrifuged at 3200 rpm at 37 °C for 15 min, the supernatant was recovered, and the bioactive compounds released in this phase were quantified. 

#### 2.3.2. Apparent Bioaccessibility 

Aqueous fractions drawn at each digestion phase (oral, gastric and intestinal) were homogenized in 100 mL of hexane and phases were allowed to separate. The organic phase (containing the tocols and minor lipophilic phytochemicals) was recovered and this procedure was repeated three times more under the same conditions. The solvent was eliminated at 40 °C and the residue was transferred to an amber bottle, sealed with nitrogen and stored at −80 °C until HPLC analysis. For this, 60 mg of the residue was dissolved in 1 mL of hexane (HPLC grade), filtered and carefully transferred to a HPLC vial wrapped in aluminum foil. Chromatographic conditions were the same as previously described and results were expressed as mg of bioaccesible tocols/100 g of sample and calculated as follows:

Apparent bioaccessibility (%) = (Total AP released per 100 g) * 100 * (Sample’s AP richness per 100 g)^−1^ (2)

where AP = Antioxidant phytochemical (T, T3, hydrophilic phenolic compounds, flavonoids or condensed tannins). Lastly, the aqueous phase resulting from the extraction of tocols at each digestive phase was used to evaluate the bioaccessibility of phenolic compounds (hydrophilic phenolic compounds, flavonoids, and condensed tannins) as previously described and spectroscopic measurements were corrected for specific controls (digestive media with no sample). Results were expressed as mg of bioaccesible hydrophilic phenolic compounds, flavonoids or condensed tannins per 100 g of raw sample. The percentage of bioaccessibility of all phenolic subgroups was calculated as described for tocols. 

### 2.4. Statistical Analysis and Chemometrics

Chemical and phytochemical composition (raw samples) and bioaccessibility (digested samples) data were expressed as the mean (n = 9) ± standard deviation (SD). After testing for data normality, one-way ANOVA followed by Tukey’s post hoc tests were performed to evaluate statistical differences (*p* < 0.05) in the chemical and phytochemical composition and in vitro bioaccessibility of antioxidant phytochemicals at each simulated digestion phase (oral, gastric, intestinal). Pearson’s product-moment correlation (r) was used to establish all possible linear correlations between all chemical/phytochemical components present in the sample’s matrices, while principal component analysis (PCA) was used to identify main components (constructs) underlying group phytochemical differences [T, T3, hydrophilic phenolic compounds, flavonoids and condensed tannins], calculating their eigenvectors and associated variance for the most significant principal components (PC; canonical variables) and plotting PC1 vs. PC2 and PC1 vs. PC3, as previously described [22]. Lastly, multiple regression analysis using the forward/reverse method and Efroymson’s algorithm was used to explain the bioaccessibility behavior of each antioxidant phytochemical (T, T3, hydrophilic phenolic compounds, flavonoids and condensed tannins) using matrices’ compositional data (total fat, MUFAs, PUFAs, total dietary fiber and each phytochemical) as explanatory factors. 

## 3. Results

### 3.1. Chemical Composition 

**Figure 2:**
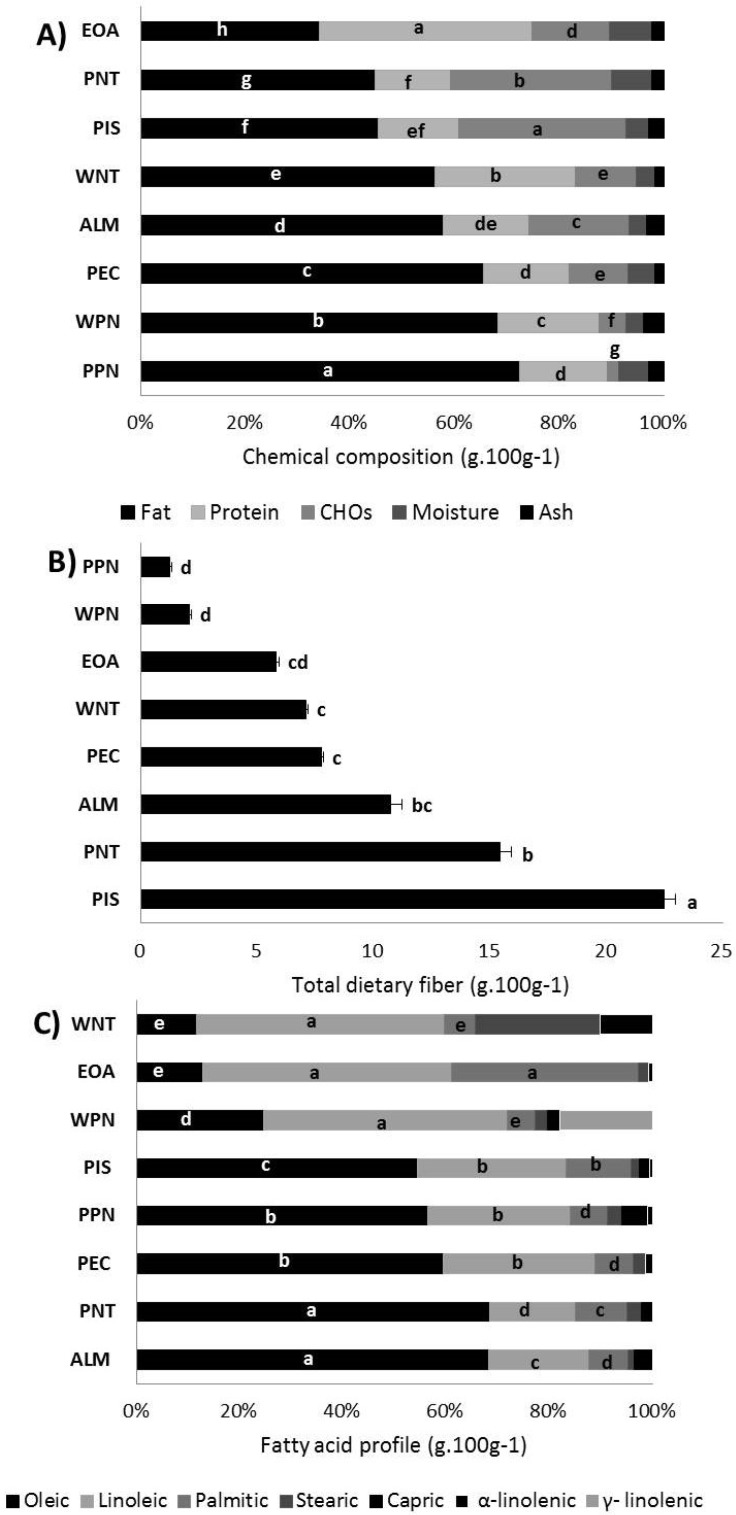
Chemical composition of edible nuts. Chemical (proximate) composition (**A**), total dietary fiber (**B**), fatty acid profile (**C**). Different letters within the same bar color series (per figure) indicate significant differences (*p* < 0.05). Almond (ALM), Emory oak acorn (EOA), pecan nut (PEC), pistachio (PIS), peanut (PNT), pink pine nut (PPT), walnut (WNT), and white pine nut (WPM).

The chemical (proximate), dietary fiber and fatty acid composition of raw samples is depicted in Figure 2. Most samples were good sources of total fat [34.2 (EOA) to 72.5 (PPN)] and protein [14.4 (PNT) to 40.5 (EOA)] and had a low moisture content (≤8.1 g 100 g^−1^; Figure 2A). Their ash, carbohydrate and total dietary fiber (Figure 2B) content ranged from 1.7 (WNT) to 3.9 (WPN), 2.2 (PPN) to 31.9 (PIS) and 1.2 (PPN) to 22.5 (PIS), respectively. Nut oils were rich (56 to 87 g 100 g^−1^) in oleic [C18:1, mainly in PIS, PPN, PEC, PNT, and ALM] and linoleic [C18:2, mainly in WNT, EOA and WPN] acids (Figure 2C). EOA was also rich in palmitic acid (C16:0, 33.2 g 100 g^−1^ oil or 11.4 g 100 g^−1^ raw nuts), while WNT was in stearic acid (C18:0, 24.0 g 100 g^−1^ oil or 13.5 g 100 g^−1^ raw nut). Total dietary fiber directly correlated (*p* ≤ 0.03) with total carbohydrates (r = 0.95) and inversely correlated with fat (r = −0.55), carbohydrates with total fat (r = −0.71) and total fat with protein (r = −0.53) and moisture (r = −0.57), respectively (Supplementary Table S1). 

### 3.2. Antioxidant Phytochemicals 

**Figure 3:**
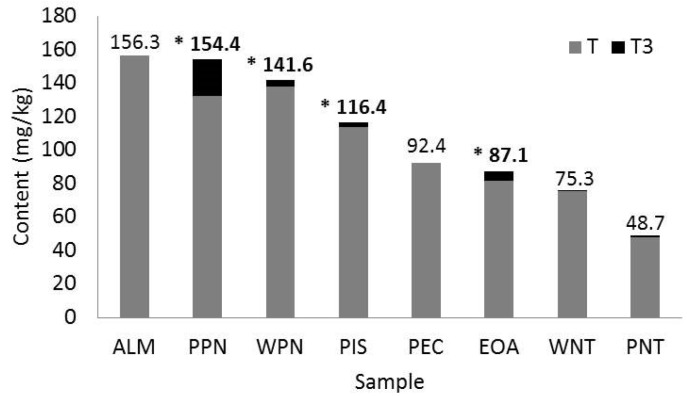
Tocols in edible nuts. Tocopherols (T), tocotrienols (T3), selected edible nut for in vitro bioaccessibility study (*). Almond (ALM), Emory oak acorn (EOA), pecan nut (PEC), pistachio (PIS), peanut (PNT), pink pine nut (PPT), walnut (WNT), and white pine nut (WPM).

The content (mg·kg^−1^) of tocols, T and T3 (Figure 3) ranged from 48.7 (PNT) to 156.3 (ALM), 48.1 (PNT) to 156.3 (ALM) and 0 (ALM, PEC) to 22.1 (PPN) mg/kg sample, respectively. Also, a sample-specific T and T3 isoform richness was also found (Table 1): ALM (αT), EOA (β, γ δT3), PEC (βT), PIS (γT, T3), PPN (γT, αT, T3), WPN (γT), WNT (γ,δT). Total tocols directly correlate (Supplementary Table S1) with total T (r = 0.98, *p* < 0.0001), ash (r = 0.77, *p* = 0.0005) and moisture (r = −0.54, *p* = 0.032), T with ash (r = 0.79, *p* = 0.0003) and T3 with total carbohydrates (r = −0.51, *p* = 0.045). On the other hand, the assayed oils were poor sources of carotenoids (range 0.01 (ALM) to 0.38 (WNT) mg·kg^−1^; Table 2)—a fact that inversely correlate with the amount of ash (r = −0.62, *p* = 0.011) in samples (Supplementary Table S1). 

The amount of lipophilic phenolic compounds (mg GAE.100 g^−1^ oil; Table 2) ranged from 3.3 (EOA) to 1219 (PPN), correlating with total fat (r = 0.93, *p* ≤ 0.0001), moisture (−0.60, *p* = 0.015), tocol (r = 0.62, *p* = 0.011), T (r = 0.58, *p* = 0.019), protein (r = −0.59, *p* = 0.016) and total carbohydrates (r = −0.55, *p* = 0.026; Supplementary Table S1).

It is noteworthy that EOA contained virtually no lipophilic phenolic compounds. Defatted flours contained substantial amounts of hydrophilic phenolic compounds (mg GAE.100 g^−1^ raw sample) ranging from 533 (PPN) to 12,896 (EOA), while their content of total flavonoids and condensed tannins ranged from 142 (WPN) to 1833 (EOA) and from 14 (PPN) to 460 (EOA), respectively (Table 2).

Hydrophilic phenolic compounds, flavonoids and condensed tannins inversely correlate with lipophilic phenolic compounds (r < −0.67, *p* = 0.004) and directly to each other (r ≥ 0.83, *p* ≤ 0.0001; Supplementary Table S1). Hydrophilic phenolic compounds correlate with fat (r = −0.71, *p* = 0.002), protein (r = 0.82, *p* = 0.0001) and moisture (r = 0.69, *p* = 0.003), flavonoids with protein and moisture (r ≥ 0.69, *p* = 0.003) and condensed tannins with fat (r = −0.69, *p* = 0.003) and moisture (r = 0.51, *p* = 0.045). Lastly, it is noteworthy that total dietary fiber did not significantly correlate (*r* = −0.47 to 0.36; *p* > 0.05) with the amount of any antioxidant phytochemical assayed (Supplementary Table S1).

### 3.3. Principal Component Analysis (PCA)

**Figure 4:**
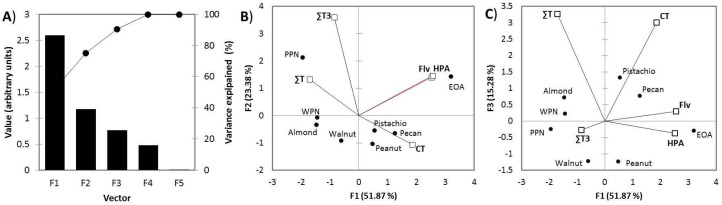
Principal component analysis (PCA). Scree (**A**) and bi-plots (**B**,**C**). Condensed tannins (CT), hydrophilic phenolic compounds (HPA), flavonoids (Flv)), monounsaturated (MUFAs) and polyunsaturated (PUFAs) fatty acids, tocopherols (T), and tocotrienols (T3). Emory oak acorn (EOA), and pink (PPN) and white (WPN) pine nut.

According to Figure 4A, three principal components (PC) explained 90.5% (PC1 (51.87%), PC2 (23.38%), PC3 (15.28%)) of the variance associated with the antioxidant phytochemical profile of all samples 

PC1 vs. PC2 explained a higher variance (75.3%; Figure 4B) than PC1 vs. PC3 (67.2%; Figure 4C). However, the spatial distribution of nuts (samples) and variables (T, T3, hydrophilic phenolic compounds, flavonoids and condensed tannins) was more differentiated in PC1 vs. PC3, reflecting a sample-specific phytochemical enrichment as binary combinations: ALM and WPN (T), PPN and WNT (T3), PIS and PEC (flavonoids, condensed tannins), EOA and PNT (hydrophilic phenolic compounds). Since PPN (22.1) > EOA (5.7) > WPN (3.6) > PIS (3.0) were also the best sources of T3 (Figure 3), these samples were selected for the in vitro bioaccessibility study.

### 3.4. Apparent Bioaccessibility

**Figure 5:**
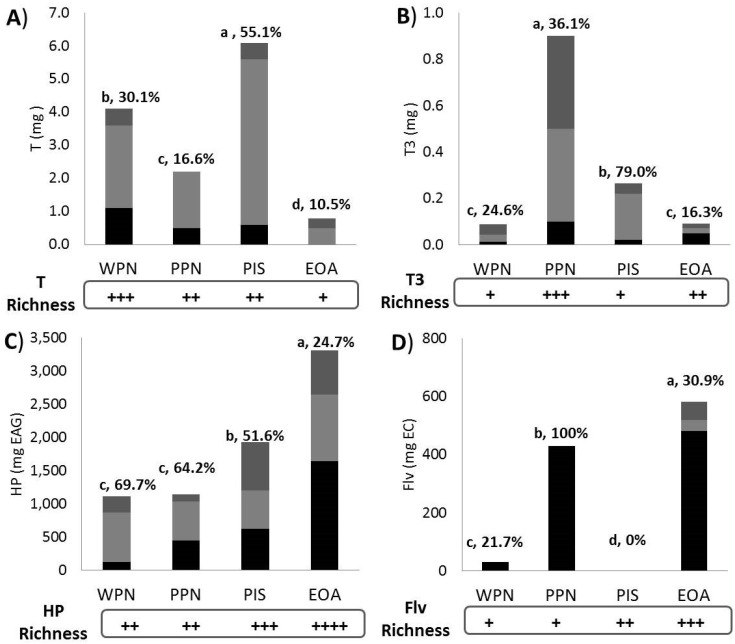
In vitro bioaccessibility of antioxidant phytochemicals from selected edible nuts. Label: Different superscript letters indicate statistical differences in the amount released from 100 g of edible nut and the percentage of in vitro bioaccessibility of tocopherols (T, (**A**)), tocotrienols (T3, (**B**)), hydrophilic phenolic compounds (HP, (**C**)) and flavonoids (FLV, (**D**)) at the simulated oral (black), gastric (light grey) and intestinal (dark grey) levels. Phytochemical richness score (see Table 1 and Table 2 for absolute values): low (+), intermediate (++), high (+++) and very high (++++). White (WPN) and pink (PPN) pine nut, pistachio (PIS), and emory oak acorn (EOA).

Figure 5 shows the in vitro bioaccessibility of T (5A), T3 (5B), hydrophilic phenolic compounds (5C) and flavonoids (5D) at the simulated oral (black), gastric (light grey) and intestinal (dark grey) levels. 

The apparent bioaccessibility (%) of tocols ranged from 10.5% (EOA) to 55.1% (PIS) for T and 16.3 (EOA) to 79.0 (PIS) for T3, respectively. With few exemptions, T and T3 were more likely to be released under simulated oral (mild alkaline) and gastric (acidic) conditions. Hydrophilic phenolic compounds were mostly released as oral > gastric > intestinal but their apparent bioaccessibility was matrix-dependent [WPN (69.7%) > PPN > PIS > EOA (24.7%)] in such a way that the higher the level of enrichment, the lower the apparent bioaccessibility. The apparent bioaccessibility of flavonoids was PPN (100%) > EOA (30.9%) > WPN (21.7%) > PIS (0%), while that of condensed tannins was much more related to nut enrichment pattern [data not shown; EOA (94.4%) > PIS (79.1%) > PPN (50.6%) > WPN (0%)]. 

Lastly, according to Table 3, the apparent bioaccessibility of T seems to be poorly but significantly (*Model 2*: R^2^ = 0.1, *p* < 0.03) related to samples’ total fat (β = −0.01) and MUFAs (β = 0.06) content, while that of T3 (*Model 2*: R^2^ = 0.94, *p* < 0.0001) was related to nut T3 content (β = 0.32) and PUFAs (β = −0.0003). 

A total of 94% (Model 2, *p* < 0.0001) of the apparent bioaccessibility of hydrophilic phenolic compounds seems to inversely depend on total fat (β = −0.50) and total dietary fiber (β = −0.27), while that of flavonoids (Model 3) and condensed tannins (Model 1) was linearly related (R^2^ ≥ 0.98, *p* < 0.001) to their own enrichment level in nuts (β = 0.10 and β = 0.008, respectively) and additionally dietary fiber (β = 0.11) and total fat (β = 0.33) just for flavonoids.

## 4. Discussion

Several epidemiological studies and intervention trials support the health benefits of regular nut consumption [3,23,24,25,26]. For example, their twice a week intake reduces the relative risk (RR, CI_95%_) for heart failure (0.80, 0.67–0.97) and atrial fibrillation (0.82, 0.68–0.99), while one serving per day reduces the risk for ischemic heart disease, cardiovascular disease and all-cause mortality (≤0.83, 0.76–0.91), when compared to low or no intake [24,25]. The health benefits of nuts have been attributed to one or more nutraceutical phytochemicals including unsaturated fatty acids, tocols, sterols, and phenolic compounds, to name a few [26,27]. However, the ultimate bioactivity of such phytochemicals is partially determined by their GI bioaccessibility and metabolism. In the GI tract, bioactive phytochemicals establish transient molecular associations with other molecules of both the food matrix (e.g., dietary fiber, fats) and GI macromolecules (e.g., mucin and enzymes) [7,9,10,11,12,13], limiting their luminal releasability and absorption at the small bowel [5,6,8]. Here, we systematically studied the chemical composition and in vitro bioaccessibility of certain antioxidant phytochemicals (tocols and phenolic compounds) from one legume (PNT), five conventional (ALM, PEC, PIS, PPN, WNT) and two non-conventional (PPN, EOA) tree nuts, searching for structural determinants present in these food matrices that may explain the in vitro bioaccessibility of the aforementioned antioxidants.

### 4.1. Chemical Composition 

All edible nuts assayed in this study were good (total fat and protein) and moderate (total dietary fiber) sources of macronutrients. Such a composition is very similar to that reported by other authors [23,26,27] and standard values from the USDA’s Food and Nutrient Database for Dietary Studies 5.0 [28], except for PPN and EOA which were not reported in these references. Before going any further, it should be stated that peanuts are not tree nuts but legumes, but they are commercially considered as such and they are commonly included in chemical compositional studies of edible nuts. The chemical (proximate) profile of PPN was similar to several *P. cembroides* accessions from Hidalgo and Chihuahua, Mexico [29]. *Quercus* acorns from two Mediterranean species (*Q. ithaburensis, Q. calliprinos*) are rich (g 100 g^−1^) in carbohydrates (42–79) and dietary fiber (13–52) but they are moderate sources of protein (2–5), fat (0.8–3.1) and ash (1.8–3.2) [30], while acorns from *Q. robur* have a moisture, ash and protein content ranging from 2.0–7.9, 2.1–8.4 and 4.2–14.1, respectively [31]; such a macronutrient composition differs from that found for EOA, which, to our knowledge, has not been reported previously. Also, the moisture content of all nuts was within the range reported by Venkatachalam and Sathe [27] and their expected low water activity (*Aw*) reduces the odds for microbial spoilage and biochemical deterioration. 

All samples were energy-dense (529–728 kcal·100 g^−1^ or 2215–3046 kJ·100 g^−1^), but they can be considered “nutritious” due to their dietary fiber and unsaturated fatty acid contents [26,27]. Dietary fiber is a complex system of non-digestible carbohydrates and lignin with graded levels of water solubility. Soluble and insoluble dietary fiber is present in different ratios in plant-based foods and according to the USDA nutrient database PIS > ALM, PNT > PEC> WNT > WPN rank as sources of total dietary fiber [23,28], just as reported here (Figure 2B). It is noteworthy that heavy tree nut consumers (44.3 g·d^−1^) are more likely to increase their daily intakes of dietary fiber (+8.5 g) and potassium (+0.84 mg) when compared to non-consumers (3.3 g·d^−1^; *p* < 0.0001), although their total fat consumption tends to increase as well [23,32]. An inverse dose–response relationship between the regular intake of soluble and insoluble dietary fibers with many non-communicable chronic diseases (NCCDs) been extensively documented [33]; however, studies comparing the specific effects of edible nut dietary fiber with that coming from other plant sources are very scarce. For instance, there is an inverse relationship between fiber intake from edible nuts with a body weight change (β = −2.01, *p* < 0.05) but not cardiovascular risk in Tehrani subjects [34].

All samples were also good sources oleic (C18:1)/linoleic (C18:2) fatty acids (≥56 g 100 g^−1^ oil), as reported by other authors [26,27]. Considering that PPN and WPN were also the best sources of fat (73 and 68 g 100 g^−1^), one hundred grams of these tree nuts will provide 41.2 and 31.3 g of oleic and linoleic acids, respectively. Oleic acid exerts anti-inflammatory, cyto-protective and microbicide action, enhances enteral drug absorption and attenuates autoimmune diseases [35], while linoleic acid is an important constituent of cell membranes and a precursor of arachidonic acid (conversion rate <1%) and prostaglandins, although its role on systemic inflammation remains controversial [36]. Data from the *Alpha Omega Cohort* indicate that an isocaloric replacement of SFA/trans fatty acids with MUFA/PUFA reduces the risk of morbidity (healthy subjects) and mortality (patients with cardiac disease) in a dose-dependent manner [37]. WNT and WPN were good sources of α-linolenic and γ-linolenic acid, respectively, a dietary deficiency of which may result in cardiovascular syndromes, hypertension, clotting abnormalities, inflammation, and diabetes mellitus [38]. Also, EOA was the richest source of palmitic acid, which plays a fundamental physiological role preserving the physical integrity of cell membranes, participates in protein palmitoylation and palmitoylethanolamide (C_18_H_37_NO_2_; a nuclear factor agonist) biosynthesis, and surfactant activity in lungs [39]. 

Lastly, the assayed nuts can also be considered moderate sources of good-quality protein and inorganic nitrogen [27]. A wide range of protein contents (14–41 g 100 g^−1^) was found in our study, being PNT and EOA the lowest and highest sources. According to Venkatachalam and Sathe [27], edible nuts are good sources of hydrophobic (37–45%) and acidic (28–33%) amino acids and do not exhibit trypsin inhibitory, proteolytic and hemagglutinating activity, while some tree nuts are also good sources of anti-hypertensive peptides [40].

### 4.2. Antioxidant Phytochemicals 

Edible nuts contain several types of phytochemicals with antioxidant activity, including tocols, carotenoids, and phenolic compounds. The concerted action of all these phytochemicals confers a total antioxidant capacity [as micromoles of Trolox equivalents per serving (26.8 g)] within the range of 20 (pine nuts) to 5095 (pecans), comparable to other antioxidant-rich fruits such as tomatoes (415), beets (1886) and blackberry (7701) [27]. 

Tocols are present in many plant foods but their richness and molar distribution in edible nuts is quite unique [19]. In this study, tocol concentration (mg·kg^−1^) ranged from 48.7 (PNT) to 156.3 (ALM), although these values are much lower than those reported by Alasalvar and Bolling [26] for all but PPN and EOA samples that were not studied by these authors. It is noteworthy that the natural distribution and amount of phytochemicals in edible nuts, including tocols, greatly depends on the fruit type, cultivar, pre- and post-harvest procedures and storage conditions [1,2,41]. On the other hand, the top four samples with the highest content of T were ALM, PPN, WPN and PIS (range 113–156 mg·kg^−1^), while the best sources of T3 were PPN, EOA, WPN and PIS (3–22 mg·kg^−1^). ALM is known to be rich in αT [23], which has been used to prevent and/or treat neurological and immunocompromised disorders, cancer and cardiovascular diseases and it accumulates in non-hepatic tissues (heart and lung) with a high mitochondrial activity that leads to high free radical production [1,2]. Also, WPN, PPN, PIS, WNT, and PEC are rich in αT and γT [26,41,42]—isoforms with proven anti-cancer properties [1].

Tocopherols are effective radical scavengers [1,2]. According to Mukai et al. [43], the relative reaction rate in micellar dispersions of αT, βT, γT and δT is 100:21:20:2.9, correlating with their biopotency for rat resorption (fetal) and hemolysis (adult) and chicken muscle dystrophy. However, T3 possess a higher antioxidant and anti-inflammatory activity than αT [2,18]. The cardio protective effects of T3 is attributed to antioxidant, hypolipidemic, anti-hypertensive and anti-atherogenic mechanisms [44] but they also exert anti-proliferative, cytotoxic, and pro-apoptotic effects in an isoform-specific (γT3, δT3) manner; such bioactivities are crucial in the treatment of breast, lung, prostate, pancreas, bladder, colon-rectum and liver cancer [1,2]. Also, the content of δT3 in PIS was within the range (0.02–2.69 mg·kg^−1^) reported by other authors [42,45] although all other isoforms were more abundant than δT3. Lastly, βT3 was only detected in WNT and, to our knowledge, T3 content in WPM, PPN, and EOA is reported for the first time. 

Carotenoids are hydrophobic isoprenoid polyenes classified as precursors (e.g., α, β-carotene and β-cryptoxanthin) and non-precursors (e.g., lycopene) of vitamin A (retinol and retinyl esters). More than forty carotenoids are present in the human diet and their bioaccessibility is very high [46]. However, peanuts and tree nuts are not considered good sources of carotenoids [19,26,28]. In this study, WNT > PIS > EOA > PEC were the best sources of carotenoids (0.15–0.38 mg·kg^−1^), with values that coincide with those reported by other authors [26,41,42,47]; however, such carotenoid contents are substantially lower than that of carrots, mangoes or squash [48]. For this and other analytical reasons, the bioaccessibility of carotenoids from the studied samples was not evaluated. 

Phenolic compounds are plant secondary metabolites derived from the phenylpropanoid and polyketide pathways. They are structurally diverse, including monomers (e.g., flavonoids) and polymers (e.g., condensed tannins), have a higher antioxidant capacity than carotenoids and tocols and are highly labile to food processing [49]. Their GI bioaccessibility, bioequivalence and plasma half-life [6,22] are crucial for their further anti-inflammatory, anti-adiposity, antidiabetic and anti-cancer action, to name just a few bioactivities [3,4]. In several systematic reviews [26,41,42,47,49,50], it has been reported that hydrophilic phenolic compounds range from 0.1–3673 mg 100 g^−1^ or 68–2016 mg GAE and, except for PPN and EOA, the values reported here tend to be higher even for the same edible nut. Many factors could explain this difference including fruit cultivar, pre/post-harvest handling and storage conditions [41] and the fact that raw (unprocessed) samples, as used in this study, often show a higher phenolic content than processed ones (e.g., artificial drying, salting, and flavoring). 

The antioxidant capacity of the assayed edible nuts directly correlates (R^2^ ≥ 0.97) with their richness in hydrophilic phenolic compounds and flavonoids [51]. The content of total hydrophilic phenolic compounds and flavonoids in PEC is similar to that reported in a previous study [52] but higher than those reported in the USDA Food and Nutrient Database [28] and for another Mexican PEC [23,24]. The content of total hydrophilic phenolic compounds and flavonoids in WPN and PPN, despite being lower than the other samples assayed here, seems to be higher than those reported by Valero-Galvan et al. [29] for several pine nut accessions from Hidalgo and Chihuahua (Mexico). It is noteworthy that EOA had 2.2 (PEC) to 23.2 (PPN) times more hydrophilic phenolic compounds than any other sample analyzed in this study. Total hydrophilic phenolics in certain *Quercus* spp. acorns range between 406–2257 mg 100 g^−1^ [53,54,55], although information on EOA has not been reported before. 

Lipophilic phenolic compounds play a pivotal role in plant defense and exert several bioactivities besides their antioxidant action. However, their content in plant foods is normally lower than hydrophilic phenolic compounds and procedures for their isolation and purification are generally laborious [56]. It is noteworthy that the amount of phenolic compounds in edible nuts is commonly quantified in hydrophilic extracts using polar solvents such as methanol, ethanol and, acetone (Pubchem XLogP3-AA: ~ −0.3), not extracting many hydrophobic phytochemicals. Here, antioxidant phytochemicals present in the oily phase were extracted with isobutanol (XLogP3-AA: 0.8) and quantified [20]. The hydrophilic/lipophilic phenolic ratio was ≤0.9 (ALM, PPN), 1–5 (WPN, WNT, PIS, and PEC) and 8.5 (PNT), while EOA practically did not have lipophilic phenolic compounds. Although the chemical nature of lipophilic phenolic compounds was not analyzed, carotenoids and the chromanol ring of tocols reacts with Folin–Ciocalteu reagent [20] and so, a significant amount of lipophilic phenolic compounds could be indeed tocols or carotenoids. More studies are needed to identify the individual hydrophilic and lipophilic phenolic fingerprint in sample sub-fractions (oil and defatted flours), to understand the extent to which they contribute to the total antioxidant capacity of edible nuts.

A wide variation in monomeric and polymeric flavonoids (condensed tannins) was observed in this study. Among samples, EOA was the richest source (1833 and 460 mg EC.100 g^−1^) with 12 and 32 times more flavonoids and condensed tannins than the lowest counterparts (WPN and PPN), respectively. To date, there are no reports on the phenolic composition of EOA but ethnographic reports provide qualitative insights as to its content of condensed tannins: (A) EOA has a protein content of 54%, but the in vitro protein digestibility is 27% [57], suggesting either a low-quality and amino acid imbalanced protein in EOA or that protease inhibitors (including condensed tannins) are present in substantial amounts in EOA’s matrix (the most reasonable explanation); (B) Raw edible EOA is commercialized in popular markets form northern Mexico as sweet but astringent foods, the main taste attribute of which is regionally defined as “agarroso” (rough, astringent) due to its condensed tannins [58].

PEC and PIS were also excellent sources of flavonoids and condensed tannins (mean ~686 and 338 mg EC.100 g^−1^) followed by PNT (406 and 164 mg EC.100 g^−1^). According to Bollling et al. [41], flavan-3-ols, flavonols, and anthocyanins are the main flavonoids in tree nuts, but their natural distribution is fruit-specific [WNT (anthocyanins), ALM (flavonols, flavan-3-ols,), PEC (flavan-3-ols, anthocyanins), PIS (flavan-3-ols, isoflavones)]. PEC > PIS are also sources of condensed tannins mostly of six to 10 monomers [41]. Condensed tannins (a.k.a. proanthocyanidins) not only have a high molecular weight but also sufficient hydroxyl groups attached to several phenolic rings, conferring them the ability to build-up complexes with proteins, minerals, and dietary fiber [59] and improving their anti-cancer properties as compared to monomeric flavonoids [60]. Also, PEC, PIS, and PEC commonly rank high in antioxidant capacity assays when compared to other edible nuts [51], so their preventive effect against the oxidative stress associated with other NCCDs can be assumed [47]. 

Lastly, PCA was used in this study to pursue meaningful relationships between the antioxidant phytochemical profile of all samples in order to reduce their data dimensionality and to choose the most representative nuts for the in vitro digestibility assay. By using this statistical tool, it was possible to identify a binary spatial distribution (differentiated eigenvectors) according to their major phytochemical identifier: T (ALM and WPN), T3 (PPN and WNT), flavonoids + condensed tannins (PIS and PEC) and hydrophilic phenolic compounds (EOA and PNT); since one of the objectives was to evaluate the bioaccessibility of T3 from edible nuts, an aspect not previously reported in the scientific literature, ALM, PPN, PIS and EOA selected for the in vitro digestion study. In a preceding study [22], we studied the phenolic profile of eight fruits rich in phenolic compounds (blackberry, blueberry, strawberry, raspberry, mulberry, pomegranate, green and red globe grapes), reporting that 93% of their variance was explained by their richness in free (61%) and bounded (32%) phenolic compounds and hierarchical analysis confirmed four clusters based on their phenolic richness and molecular diversity.

### 4.3. Apparent Bioaccessibility 

Regular consumption of oilseeds and tree nuts has been recommended by many medical organizations to prevent and even treat several NCCDs [23,24,25,26,33,42]; such benefits partially depend on the effective delivery and absorption of their phytochemicals along the GI tract. However, during this journey, molecules can be chemically modified and so, their expected bioefficacy [5,6,7,8,9,10,11,12]. The first event to overcome is the releasability of phytochemicals from its food matrix through mechanical, chemical, and enzymatic forces, event conventionally known as bioaccessibility [6,10,13]. This mechanistic transformation is not only needed to ensure the maximal luminal concentration of nut’s phytochemicals but also for a proper nutrient/xenobiotic signaling and metabolic adaptation needed for their absorption, peripheral transport and target delivery [6,61]. Several analytical methods have been developed to evaluate the apparent bioaccessibility of phytochemicals from which static and dynamic in vitro digestion methods are the most widely used [62]. While recognizing certain drawbacks (e.g., lack of homeostatic/transport mechanisms and limited responsiveness to composition and quality of foods), simple static in vitro digestion methods mimicking the GI tract features have been proposed as alternatives to in vivo experiments [63]. 

According to Bolling et al. [41], oilseeds and tree nut phytochemicals are both bioaccessible and bioavailable in humans in a fruit-specific manner. In this study, by using a previously standardized static in vitro digestion method [22] we estimate the apparent bioaccessibility of T (11–51%), T3 (16–79%), hydrophilic phenolics (25–55%), flavonoids (0–100%) and condensed tannins (0–94%) from ALM, PPN, WPN and EOA. These nuts were selected as representative samples from all eight samples, since they differed in their phytochemical enrichment pattern (see “phytochemical enrichment” boxes below each graph), but they were also the top four richest samples in T3. Data indicate that these phytochemicals were released at a specific digestive stage: oral (flavonoids and condensed tannins), gastric (T), gastric-intestinal (T3), oral-gastric-intestinal (hydrophilic phenolic compounds).

Chewing and saliva mixing (oral stage) are crucial in modifying the consistency of foods. They render boluses, the physical properties (e.g., reduced particle size and hydration) of which allow their easy passage through the larynx and down the esophagus into the stomach [64]. The extent to which edible nuts are processed at this stage has a major effect on the amount and type of phytochemicals released. In this study, the apparent bioaccessibility of flavonoids and condensed tannins from the assayed samples was high under simulated oral conditions. Particularly, the apparent bioaccessibility of flavonoids was WPN (21.7%) > PIS (0%) and for condensed tannins was PIS (79.1%) > WPN (0%), while their original content (as mg EC) was PIS > WPN (2.9 times lower) for flavonoids and PIS > WPN (7.6 times lower) for condensed tannins, respectively. Yang et al. [51] reported that flavonoids in edible nuts are found in free form (PNT, PEC, PIS, WNT), bounded (WPN) or both (ALM) Assuming that bonded flavonoids are actually condensed tannins (polymeric flavonoids), differences with our results may be due to methodological differences including sample processing (raw vs. roasted) or origin (e.g., Mexican vs. Korean WPN). As for total hydrophilic compounds, their oral releasability seemed to follow a dose (nut enrichment level)-response behavior—a fact that we previously reported for berry fruits [22]. Yang et al. [51] reported that hydrophilic compounds (as assayed by the Folin–Ciocalteu method) are mostly covalently bound to their matrix except for PEC and WNT (mostly free form) or PNT and PIS (evenly distributed), while Rocchetti et al. [65] reported that phenolic compounds in PNT, PIS, ALM, WNT and hazelnuts are mainly found in free (unbonded) form. Again, the differences found between these research groups and ours could be due to methodological differences. 

A low amount of tocols (T + T3) was released at oral conditions, as related to the original enrichment level in the assayed nuts. According to Ellis et al. [66], the seed coat of ALM is rich in non-starch arabinose-rich polysaccharides with a high amount of bounded phenolic compounds, while lipid droplets, located in more internal layers of the cotyledon, are not disrupted enough by mechanical methods or chewing. Mandalari et al. [67] later demonstrated a higher in vitro bioaccessibility of lipid, T (mainly αT) and protein (39–45%) from finely grounded but not poorly fractured ALM, a significant correlation between T’s and total lipid’s bioaccessibility and that nutrient/phytochemical bioaccessibility from un-fractured ALM is also possible but a much slower rate. In this study, ALM was not selected for the in vitro digestion assay because it was not a good source of T3. However these arguments do apply when comparing two samples with different textural profiles: PPN showed a higher fat and T3 but lower dietary fiber and total carbohydrate content than PIS and, consequently, WPN nutmeat tend to be softer (data not shown); This issue probably explains why T were more effectively extracted from WPN than PIS under simulated oral conditions (were nut cracking occurs). 

The above evidence also suggests functional compartmentalization of antioxidant phytochemicals in the assayed tree nuts. According to Liu et al. [68], the GI bioaccessibility of molecules from plant cells requires the disruption of the following: (i) the vacuolar tonoplast (vacuolar membrane), an organelle rich in polar lipids, unsaturated fatty acids and transport proteins that is crucial for protoplasmic homeostasis and to maintain the cell’s turgor pressure, (ii) the plasma membrane (amphipathic, protein-rich) and (iii) the extracellular cell wall (amphipathic, polysaccharide-rich); while the vacuolar tonoplast is rich in monomeric/hydrophobic phytochemicals (e.g., tocols), the plasma membrane and the cell wall are rich in monomeric and polymeric phenolic compounds (free + bounded), respectively [69]. It is noteworthy that vacuolar tonoplasts are pH-resistant and plasma membranes are temperature-resistant but once cell walls are disrupted by mastication and GI enzymes, the resistance of these microstructural elements totally relies on their chemical composition and size.

Regardless of the initial meal viscosity, the addition of salivary and then gastric secretions causes high dilution of the bolus [64]. This fact combined with the low gastric pH causes the structural relaxation (denaturation) of matrix macromolecules (e.g., protein and dietary fiber) and the releasability of trapped or weakly bonded small molecules. Here, a substantial amount of tocols [T (11–55%) > T3 (16–79%)] was released mostly under simulated gastric conditions. Liu et al. [70] employed a static in vitro digestion method to estimate the apparent bioaccessibility of γT from PIS observing a low apparent bioaccessibility (5–7%), while Mandalari et al. [67], by using a dynamic in vitro digestion method, reported an extremely high apparent bioaccessibility of αT, δT, γT (≥ 88%) from PIS, mostly under simulated gastric conditions. Yang and McClements [9] evaluated the influence of carrier oil type [long-chain (LCT) and medium-chain (MCT) triglycerides] on digestion and apparent bioaccessibility of emulsified αT acetate observing that after passage through oral and gastric stages, the mean particle diameter remained relatively small (d32 < 400 nm) but their electrical charge decreased appreciably under gastric conditions depending on the emulsifier [MCT (~−6 mV), LCT (~+0.5 mV)]. According to Jakobek [69], molecules surrounding emulsions could be found inside of the oil droplet, in the water phase that surrounds the oil droplet or in the interfacial region, determining their superficial polarity. Since tree nut triglycerides are made of ≥14C fatty acids [26,27,28], the simultaneous release of fatty acids and tocols from the assayed samples under gastric conditions could be causing a rapid production of stable micelles [67,69], although the presence of tocopheryl fatty acids in these samples cannot be ruled out, as found for other tree nuts such as WNT [71]. 

Total bioaccessibility of hydrophilic phenolic compounds from the assayed nuts was additive (oral > gastric > intestinal) and dose (nut enrichment)-dependent, as it happens with phenolic compounds from other fruits [22,62]. However, the amount specifically released under simulated gastric conditions was EOA > WPN > PPN, PIS—a fact surely related to their macronutrient composition (higher source): dietary fiber (ALM), fat (WPN, PPN) and protein (EOA, PIS). Our results for PIS agree with Mandalary et al. [12] as to the releasability of phenolic compounds under gastric conditions, although the absolute bioaccessibility at this stage differed; anyhow, such bioaccessibility from PIS was similar to that reported by Liu et al. [70]. However, the observed bioaccessibility of hydrophilic phenolic compounds could also be due to an enhanced pepsin activity. According to Plundrich et al. [72], the presence of PNT protein–phenolic compounds complexes enhances pepsin activity (as compared to PNT proteins alone), contributing to a much higher bioaccessibility of phenolic compounds (and possible other antioxidant phytochemicals) from high protein/low dietary fiber nuts such as EOA.

Non-covalent (e.g., with dietary fiber), hydrophobic (e.g., with proteins) and covalent (e.g., with fats) bonds between phenolic compounds and complex molecules are partially weakened by mechanical and chemical action during their oral–gastric passage, releasing mostly unbounded (trapped) antioxidant phytochemicals [6,64]. However, an additional amount is released under intestinal conditions, mostly by enzymatic action; according to Baer and Novotny [73], total digestibility (oral-to-intestinal) of proteins, fats, and carbohydrates from edible nuts is about ~78%, ~90%, and ~97%, respectively, although certain matrix components limit such digestion. In this study, the intestinal releasability of hydrophilic phenolic compounds was particularly evident, besides a small portion of T from PIS and WPN, T3 from PPN and flavonoids from EOA. Bile salts and fats (micelle components) and other GI biomolecules can ‘‘capture’’ antioxidant phytochemicals and protect them during their intestinal passage [9,11,74], targeting them for absorption (small bowel) or microbial biotransformation (large bowel) [6,13,69]; it should be pointed out that a lot of attention has been paid to the interaction of antioxidant phytochemicals with dietary fiber and pancreatic enzymes [6,13,74], although in the case of edible nuts, interactions with unsaturated fats and proteins seem to be more relevant [11,12,67]. 

### 4.4. Chemometrics

The use of spectroscopic and high through-output chromatographic techniques (e.g., HPLC-qTOF-DAD-MS/MS) to simultaneously evaluate many chemical compounds in the same sample is customary practice in food and pharmaceutical sciences; however, this practice sometimes results in a high amount of experimental data from which scientists derive specific “chemical fingerprints” of foods by means of chemometrics. Chemometric tools are commonly used to reduce the dimensionality of experimental data by using mathematical models and multivariate statistics such as principal component analysis (PCA), simple correlation (e.g., Pearson’s product-moment correlation) and multiple linear regression (MLR) [22,55]. 

In this study, Spearman’s rank correlation, a measure of the strength and direction of association existing between two continuous variables, provided preliminary information on a presumable concentration-dependent relationship between certain structural components of nut matrices and the evaluated antioxidant phytochemicals. Lipophilic compounds such as tocols (T + T3; XLogP3-AA range = 8.6–10.7) [48], carotenoids (lutein, zeaxanthin, β-carotene; XLogP3-AA = 11–13.5) [75] and lipophilic phenolic compounds showed significant correlations (either direct or inverse) with total fat and protein (lipophilic phenolic compounds), moisture (total tocols), ash (total tocols, T, carotenoids), and total carbohydrates (T3, lipophilic phenolic compounds). Conversely, polar phenolic compounds (hydrophilic phenolic compounds, flavonoids, and condensed tannins) directly or inversely correlated with hydrophilic (moisture, protein) and hydrophobic (fat, lipophilic phenolic compounds) components of nut matrices, respectively. It is noteworthy that total protein content was inversely associated with total dietary fiber and fat (mean r = −0.54, *p* = 0.03) but total dietary fiber did not correlate (r = −0.47 to 0.36; *p* > 0.05) with the amount of any antioxidant phytochemicals. Once again, this evidence further supports the functional compartmentalization of antioxidant phytochemicals in the assayed tree nuts [68,69]. 

We further investigated which matrix components were more closely related to the absolute (oral + gastric + intestinal) in vitro bioaccessibility of antioxidant phytochemicals by using MLR. Mathematical models suggested a statistically significant correlation, either direct (+) or inverse (-), of certain matrix components with the bioaccessibility of antioxidant phytochemicals (*p*≤ 0.03), in a structure dependent manner: T [total fat (-) and MUFAs (+); R^2^ = 0.1], T3 [nut’s T3 (+) and PUFA (-) contents; R^2^ = 0.94], hydrophilic phenolic compounds [total fat (-), dietary fiber (-); R^2^ = 0.94], flavonoids [nut’s flavonoid (+), dietary fiber (+), and total fat (+) contents; R^2^ = 0.99] and condensed tannins [nut’s condensed tannins (+); R^2^ = 0.98]. It is noteworthy that dietary fiber explained an additional 4%, 9% and 16% of the variance associated with T3, flavonoids and hydrophilic phenolic compounds, respectively. These findings allow us to conclude that fats (total, MUFA or PUFA) could have an essential role in the simultaneous emulsification and bioaccessibility of tocols and flavonoids (to a lesser extent), while dietary fiber has a decisive role in the bioaccessibility of hydrophilic phenolic compounds and flavonoids but not condensed tannins. 

Verkempinck et al. [76] reported that MUFAs > PUFAs facilitate a faster micelle assembly due to the fact that the presence of one or more un-saturations in fatty acid lead to a more complex micellar structure, lesser amphipathic nature and more resistance to hydrolysis by pancreatic lipase. Since T3 isoforms [α, β, γ, δ] differ from their T counterparts in a higher number of unsaturated bonds in their phytil tail [2], this partially explains their differential affinity toward PUFAs and MUFAs, respectively. The slower formation of T3-containing micelles may also help to explain why T3 > T were released at the intestinal stage, while most T were rapidly released at oral and gastric stages. Also, T bioaccessibility was negatively related to total fat, which was somehow unexpected; however, the positive effect of MUFAs indicates that the kind of fatty acid, more than total fat, is important for the proper emulsification of these lipophilic molecules. 

On the other hand, the bioaccessibility of total hydrophilic phenolic compounds and flavonoids were differently influenced by the matrix’s total fat and dietary fiber. According to Jakobek and Matić [13], dietary fibers control the site-specific delivery of phenolic compounds in the upper or lower parts of the digestive tract. It is known that dietary fiber acts negatively on nutmeat’s fat releasability, reducing the interaction of triacylglycerides with enzymes and bile salts, reducing fatty acid releasability and micelle formation [67,69]. An interesting finding was that the dietary fiber–phenolic relationship, which by the way was not evidenced by Spearman’s rank correlation (simple regression analysis), was negative for total hydrophilic phenolic compounds but positive for flavonoids, as evidenced by multiple regression analysis. Some authors [6,11,49,56] point out that high-fat food matrices favor the bioaccessibility of both hydrophilic and lipophilic phenolic, although this fact has been scarcely studied in peanuts and tree nuts [12,52,70] and so the nature of such a structural relationship deserves further investigation. 

Lastly, the apparent bioaccessibility of condensed tannins only correlated with their enrichment level in nuts, while no other matrix component explained their releasability. The same stage-dependent bioaccessibility of condensed tannins (oral > gastric, intestinal) has been reported in other plant sources such as spent coffee grounds [77] and *Moringa oleifera* seed flour [78]. As previously mentioned, condensed tannins have the ability to form complexes with proteins, minerals, and dietary fiber [59,69] in such a way that supramolecular complexes can also be built up, reducing tannin emulsification in GI juices and their subsequent extraction and quantification. It should be noted that condensed tannins are also effective enzyme inhibitors [59,74] which also reduces the possibility of degrading the food matrix to achieve their own bioaccessibility. Nevertheless, the maximum in vivo bioaccessibility of condensed tannins is achieved under colonic conditions by the concerted action of several microbial enzymes including tannase [79] and β-glucosidase [80]—a fact that could not be proven in this study.

## 5. Conclusions

Peanuts and tree nuts are rich sources of antioxidants phytochemicals with a wide range of bioactivities. However, their ultimate health effects are partially determined by their GI bioaccessibility. Results from this study suggest that such releasability depends on several chemical components present in nut matrices from which dietary fiber and monounsaturated fats emerged as the most significant factors, besides their original enrichment level in the tested nuts. The study also showed that most antioxidants were released under oral and gastric conditions but bioaccessibility under intestinal conditions is also important for some of them (e.g., T and hydrophilic phenolic compounds). Further studies are needed to evaluate the nature of all chemical interactions established between these antioxidant phytochemicals and certain macromolecules present in both nut matrices and GI biomolecules.

## Figures and Tables

**Table 1:** Tocol isoforms in edible nuts ^1^.

Edible Nut	α	β	γ	δ
**Tocopherols (T)**				
Almond (ALM)	124.9 ± 4.8 ^a^	16.8 ± 0.4 ^a^	14.5 ± 0.0 ^d^	--
**Emory oak acorn (EOA)**	10.0 ± 0.1 ^e^	15.2 ± 0.6 ^b^	55.9 ± 4.7 ^c^	0.2 ± 0.0 ^c^
Pecan (PEC)	17.1 ± 0.1 ^d^	18.0 ± 0.3 ^b^	56.9 ± 2.8 ^c^	0.02 ± 0.0 ^c^
**Pistachio (PIS)**	15.2 ± 0.1 ^d^	15.7 ± 0.4 ^a^	82.1 ± 6.2 ^b^	0.4 ± 0.0 ^b^
Peanut (PNT)	13.4 ± 0.4 ^d,e^	11.4 ± 0.1 ^c^	22.8 ± 0.7 ^d^	0.5 ± 0.0 ^b^
**Pink pine nut (PPN)**	43.9 ± 2.3 ^b^	4.5 ± 0.04 ^d^	83.6 ± 1.7 ^b^	0.3 ± 0.0 ^c^
**White pine nut (WPN)**	24.2 ± 0.6 ^c^	5.2 ± 0.0 ^d^	108.6 ± 1.3 ^a^	0.2 ± 0.0 ^c^
Walnut (WNT)	--	15.4 ± 0.2 ^b^	58.1 ± 1.3 ^c^	1.7 ± 0.1 ^a^
**Tocotrienols (T3)**				
ALM	--	--	--	--
**EOA**	0.2 ± 0.0 ^c^	0.6 ± 0.1 ^a^	1.7 ± 0.1 ^b^	3.3 ± 0.1 ^a^
PEC	0.3 ± 0.0 ^c^	0.1 ± 0.0 ^c^	0.3 ± 0.2 ^d^	0.3 ± 0.0 ^b^
**PIS**	0.4 ± 0.1 ^c^	0.3 ± 0.1 ^b^	2.0 ± 0.1 ^a^	0.2 ± 0.0 ^b^
PNT	--	0.5 ± 0.0 ^b^	0.1 ± 0.0 ^e^	--
**PPN**	20.4 ± 1.0 ^a^	0.2 ± 0.0 ^b,c^	1.3 ± 0.1 ^a,b^	0.3 ± 0.0 ^b^
**WPN**	2.2 ± 0.3 ^b^	0.1 ± 0.0 ^c^	1.1 ± 0.1 ^c^	0.2 ± 0.0 ^b^
WNT	--	0.1 ± 0.0 ^c^	--	--

^1^ Values are expressed as the mean (mg/kg) ± SD; different superscript letters within each column per parameter (T or T3) indicate statistical differences between samples (*p* < 0.05); below the detection limit (--), tree nuts selected for the in vitro digestion assay are depicted in **bold**.

**Table 2:** Phenolic compounds and carotenoids in edible nuts ^1^.

Edible Nut	HP (mg GAE)	Flv (mg CE)	CT (mg CE)	LP (mg GAE)	CAR (mg/kg)
Almond (ALM)	727 ± 37 ^e^	188 ± 14 ^d^	85 ± 2 ^d^	812 ± 57 ^b,c^	0.01 ± 0.07 ^d^
**Emory oak acorn (EOA)**	12,896 ± 390 ^a^	1833 ± 40 ^a^	460 ± 16 ^a^	3.3 ± 11 ^e^	0.16 ± 0.01 ^b^
Pecan (PEC)	3990 ± 111 ^b^	814 ± 18 ^b^	348 ± 23 ^b^	866 ± 33 ^b^	0.15 ± 0.07 ^b^
**Pistachio (PIS)**	2530 ± 30 ^c^	557 ± 41 ^c^	328 ± 18 ^b^	555 ± 5 ^d^	0.17 ± 0.06 ^b^
Peanut (PNT)	2715 ± 77 ^c^	406 ± 30 ^c^	164 ± 10 ^c^	318 ± 2 ^e^	0.02 ± 0.01 ^d^
**Pink pine nut (PPN)**	533 ± 74 ^e,f^	231 ± 17 ^d^	14 ± 5 ^e,f^	1219 ± 56 ^a^	0.05 ± 0.00 ^d^
**White pine nut (WPN)**	818 ± 20 ^e^	142 ± 7 ^d^	38 ± 8 ^e^	746 ± 13 ^c^	0.04 ± 0.00 ^d^
Walnut (WNT)	1407 ± 28 ^d^	169 ± 3.3 ^d^	115 ± 8 ^d^	755 ± 23 ^c^	0.38 ± 0.05 ^a^

^1^ Values are presented as the mean ((value)/100 g) ± SD (n = 9). Different letters within the same column indicate statistical differences (*p* < 0.05); hydrophilic (HP) and lipophilic (LP) phenolic compounds, flavonoids (Flv), condensed tannins (CT), total carotenoids (CAR), gallic (GAE) and catechin (CE) equivalents. Tree nuts selected for in vitro digestion evaluation (**bold**).

**Table 3:** Multivariate regression analysis of phytochemical in vitro bioaccessibility ^1^.

Model	Matrix’s Phytochemical ^1^	β_1_	β_2_	β_3_	R^2^	*p*
T-BA						
1	TF	0.01	-	-	0.09	>0.05
2	TF + MUFAs	−0.014	0.057	-	0.10	<0.03
T3-BA						
1	PUFAs	−0.002	-	-	0.36	<0.03
2	PUFAs + T3	−0.0003	0.32	-	0.94	<0.0001
3	PUFAs + T3 + DF	0.004	0.39	0.009	0.98	<0.0001
HP-BA						
1	TF	−0.52	-	-	0.78	<0.0001
2	TF + DF	−0.50	−0.27	-	0.94	<0.0001
Flv-BA						
1	Flv	0.022	-	-	0.36	<0.02
2	Flv + DF	0.019	−0.036	-	0.45	<0.03
3	Flv + DF + TF	0.10	0.11	0.33	0.99	<0.001
CT-BA						
1	CT	0.008	-	-	0.980	<0.001
2	CT + DF	0.008	−0.008	-	0.989	<0.002

^1^ Initial content in fruits (Table 1 and Table 2); bioaccessibility (BA), condensed tannins (CT), total dietary fiber (DF), flavonoids (Flv), hydrophilic phenolic compounds (HP), mono- (MUFAs) and poly- (PUFAs) unsaturated fatty acids, tocopherols (T), tocotrienols (T3), and total fat (TF).

## Supplementary Materials

The following are available online at https://www.mdpi.com/2072-6643/11/10/2303/s1, Table S1: Pearson’s product moment correlations.
