# Supplementary material for: Chemical Composition and In Vitro Bioaccessibility of Antioxidant Phytochemicals from Selected Edible Nuts
Source: Nutrients. 2019 Sep 27;11(10):2303. doi: 10.3390/nu11102303 (PMC6836022; doi:10.3390/nu11102303)
Supplement: Supplementary file 1 [file nutrients-11-02303-s001.pdf]

Table 1. Pearson's product moment correlations[1].

	**1**	**2**	**3**	**4**	**5**	**6**	**7**	**8**	**9**	**10**	**11**	**12**	**13**	**14**
Moisture (1)	1.00													
Protein (2)	0.37	1.00												
Fat (3)	**-0.57**	**-0.53**	1.00											
Ash (4)	-0.44	-0.33	0.33	1.00										
Carbohydrates (5)	0.24	-0.22	**-0.71**	-0.12	1.00									
SDF (6)	0.04	-0.35	**-0.55**	-0.07	**0.95**	1.00								
Tocopherols (7)	-0.63	-0.29	0.55	**0.79**	-0.38	-0.25	1.00							
Tocotrienols (8)	0.23	0.00	0.38	0.19	**-0.51**	-0.47	0.31	1.00						
Tocols (9)	**-0.54**	-0.27	0.58	**0.77**	-0.45	-0.32	**0.98**	0.47	1.00					
Carotenoids (10)	-0.19	0.46	-0.20	**-0.62**	-0.05	0.04	-0.42	-0.25	-0.43	1.00				
LP(11)	**-0.60**	**-0.59**	**0.93**	0.23	**-0.55**	-0.36	**0.58**	0.44	**0.62**	-0.09	1.00			
HP(12)	**0.69**	**0.82**	**-0.71**	-0.38	0.10	-0.05	-0.42	-0.07	-0.40	0.17	**-0.78**	1.00		
Flavonoids (13)	**0.69**	**0.74**	-0.66	-0.37	0.11	0.00	-0.37	-0.04	-0.35	0.14	**-0.71**	**0.98**	1.00	
CT (14)	**0.51**	0.49	**-0.69**	-0.45	0.38	0.36	-0.44	-0.31	-0.47	0.29	**-0.67**	**0.83**	**0.88**	1.00

[1]Condensed tannins (CT), hydrophilic (HP) & lipophilic (LP) phenolic compounds, soluble dietary fiber (SDF); statistically significant ($p<0.05$) correlations (**bold**).
